# Upregulation of ATP6V0D2 benefits intracellular survival of *Leishmania donovani* in erythrocytes-engulfing macrophages

**DOI:** 10.3389/fcimb.2024.1332381

**Published:** 2024-01-31

**Authors:** Jing Hong, Budhaditya Mukherjee, Chizu Sanjoba, Junya Yamagishi, Yasuyuki Goto

**Affiliations:** ^1^ Laboratory of Molecular Immunology, Graduate School of Agricultural and Life Sciences, The University of Tokyo, Tokyo, Japan; ^2^ School of Medical Science and Technology, Indian Institute of Technology Kharagpur, Kharagpur, India; ^3^ International Collaboration Unit, International Institute for Zoonosis Control, Hokkaido University, Sapporo, Japan

**Keywords:** *Leishmania*, macrophage, multinucleated giant cell (MGC), ATP6V0D2, iron, hemophagocytosis

## Abstract

Visceral leishmaniasis (VL) is the most severe type of leishmaniasis which is caused by infection of *Leishmania donovani* complex. In the BALB/c mouse model of VL, multinucleated giant cells (MGCs) with heavy parasite infection consist of the largest population of hemophagocytes in the spleen of *L. donovani*-infected mice, indicating that MGCs provide the parasites a circumstance beneficial for their survival. Although ATP6V0D2 is a demonstrated factor inducing the formation of hemophagocytic MGCs during *L. donovani* infection, functions of this protein in shaping the infection outcome in macrophages remain unclear. Here we evaluated the influence of upregulated ATP6V0D2 on intracellular survival of the parasites. *L. donovani* infection-induced hemophagocytosis of normal erythrocytes by macrophages was suppressed by RNAi-based knockdown of *Atp6v0d2*. The knockdown of *Atp6v0d2* did not improve the survival of amastigotes within macrophages when the cells were cultured in the absence of erythrocytes. On the other hand, reduced intracellular survival of amastigotes in macrophages by the knockdown was observed when macrophages were supplemented with antibody-opsonized erythrocytes before infection. There, increase in cytosolic labile iron pool was observed in the *L. donovani*-infected knocked-down macrophages. It suggests that ATP6V0D2 plays roles not only in upregulation of hemophagocytosis but also in iron trafficking within *L. donovani*-infected macrophages. Superior access to iron in macrophages may be how the upregulated expression of the molecule brings benefit to *Leishmania* for their intracellular survival in the presence of erythrocytes.

## Introduction

Visceral leishmaniasis (VL) is the most severe form of leishmaniasis which has estimated 30,000 new cases annually (World Health Organization, 2023) and is often fatal if not treated appropriately (World Health Organization, 2021). It is caused by the infection of vector-borne protozoan *Leishmania* species including *L. donovani* (Ld) that disseminates into internal organs such as spleen and bone marrow (World Health Organization, 2010).

Macrophages, the host cells of *Leishmania*, are a group of professional phagocytic cells that protect a body from pathogens by engulfing and degrading the foreign particles in their mature hydrolytic and oxidative phagolysosomes (Desjardins, 1995; Nguyen and Yates, 2021). In addition, they are indispensable in tissue homeostasis maintenance by eliminating aged/damaged cells including erythrocytes and recycling iron ions from these cells (Italiani and Boraschi, 2014). However, in infection of *L. donovani*, macrophages exhibit enhanced hemophagocytic activity toward undamaged erythrocytes, which can be linked to the onset of anemia in VL (Goto et al., 2017). We previously reported that *L. donovani*-infected macrophages are prompted to engulf undamaged erythrocytes and the uptake of erythrocytes is beneficial to the parasites for intracellular survival (Morimoto et al., 2019). Moreover, *L. donovani* infection also stimulates the formation of multinucleated giant cells (MGCs) which exhibit a high hemophagocytic activity (Hong et al., 2022).

In mammals, hemoglobin accounts for the largest pool of heme and iron (Hamza and Dailey, 2012) and consequently, erythrocytes are supposed to be the most abundant iron resource. On the other hand, iron acquisition is critical for intracellular survival of *Leishmania* (Laranjeira-Silva et al., 2020). For example, *L. amazonensis* lacking iron transporter LIT1 is unable to replicate in macrophages (Mittra et al., 2013), while the ferric iron reductase (LFR1) of *Leishmania* is also essential for intracellular survival (Flannery et al., 2011).

ATP6V0D2 is a subunit of vacuolar ATPase (V-ATPase) which is proposed to function as proton pump and regulates organellar acidification in mammal cells (Vasanthakumar and Rubinstein, 2020). It is also reported that ATP6V0D2 is indispensable in osteoclastogenesis as mice lacking Atp6v0d2 gene cannot form mature osteoclasts (Lee et al., 2006). We previously demonstrated that the formation of *L. donovani* infection-induced MGCs is mediated by the upregulated expression of ATP6V0D2 (Hong et al., 2022). The molecule not only functions in osteoclast and MGC formation but also affects the outcome of several infectious diseases. *Staphylococcus aureus* is the common causative organism in osteomyelitis (Lew and Waldvogel, 2004), and its infection in macrophages induces upregulation of ATP6V0D2 expression and acquisition of osteoclast-like bone resorption activity (Ren et al., 2017). On the other hand, expression of ATP6V0D2 decreases in *Salmonella typhimurium*-infected BMDMs and the reduction leads to autophagosome-lysosome fusion and higher intracellular bacterial burden (Xia et al., 2019).

In *Leishmania* infection, it is reported that in *L. amazonensis* infection *Atp6v0d2* controls *Leishmania* parasitophorous vacuole (PV) biogenesis via affecting cholesterol homeostasis whereas no influence on intracellular survival of the parasites is observed (Pessoa et al., 2019). Nonetheless, the involvement of ATP6V0D2 in the outcome of *L. donovani* infection remains unclear. Hence, in this study we explored the influence of upregulated ATP6V0D2 in the survival of *L. donovani* amastigotes by highlighting its effect on hemophagocytosis by infected macrophages as well as its involvement in the beneficial effect of hemophagocytosis to the parasites.

## Materials and methods

### Ethics statement

All animal experiments were reviewed and approved by the Animal Experiment Committee at the University of Tokyo (Approval No. P17-076 and P20-063). The experiments were performed in accordance with the Regulations for Animal Care and Use of the University of Tokyo, which were based on the Law for the Humane Treatment and Management of Animals, Standards Relating to the Care and Management of Laboratory Animals and Relief of Pain (the Ministry of the Environment), Fundamental Guidelines for Proper Conduct of Animal Experiment and Related Activities in Academic Research Institutions (the Ministry of Education, Culture, Sports, Science and Technology) and the Guidelines for Proper Conduct of Animal Experiments (the Science Council of Japan). Collection of blood from mice was performed under anesthesia with isoflurane. At the end of the experiments, mice were euthanized by exsanguination under anesthesia with isoflurane followed by cervical dislocation.

### Mice, cells and parasites

Female BALB/cA mice were purchased from Japan Clea, Tokyo, Japan. All mice were maintained under specific pathogen-free conditions. The mice were used for experiments at the age of 6–8 weeks.

Bone marrow cells were isolated from femurs and tibias of BALB/cA mice. Bone marrow-derived macrophages (BMDMs) were generated by cultivating bone marrow cells in DMEM (Wako, Japan) supplemented with 10% heat-inactivated fetal bovine serum (HI-FBS, Thermo Scientific, USA), 100 U/ml penicillin + 100 μg/ml streptomycin (Wako) and 25 ng/ml recombinant mouse macrophage colony stimulated factor (M-CSF, PeproTech, USA) for 7 days at 37°C and 5% CO_2_. The medium was changed once with fresh one on Day 4.

*L. donovani* promastigotes (MHOM/NP/03/D10, gifted from National BioResource Project at Nagasaki University (Pandey et al., 2007)) were cultured in medium 199 (Invitrogen, USA) supplemented with 10% HI-FBS at 25°C. In some experiments, *Leishmania* promastigotes were stained with CytoRed (Dojindo Laboratories, Japan). 1 × 10^7^ promastigotes were incubated in 100 μl of DMEM medium containing 50 μg/ml of CytoRed at room temperature for 30 minutes. The stained promastigotes were washed with DMEM three times and used for *in vitro* infection experiments.

### RNA interference

RNA interference was performed with the following small interfering RNAs (siRNAs): si-Control (#4390843, Invitrogen); si-ATP6V0D2 (s109716, #4390771, Invitrogen). 6 μl of 10 μM siRNA were incubated with 9 μl of Lipofectamine RNAiMAX (Invitrogen) in DMEM for 5 minutes. 2 × 10^6^ cells of Day 6 BMDMs were transfected with the mixture for 24 hours. For *Leishmania* infection group, the transfected BMDMs were incubated with *L. donovani* 24 hours after transfection.

### Opsonization and fluorescent labeling of RBCs

Preparation of the opsonized RBCs were performed by incubating 2 × 10^7^ murine erythrocytes with 0.5 μg of monoclonal anti-mouse red blood cell (RBC) antibodies (HM1120-FS, Hycult Biotechnology B.V., Netherlands) in DMEM for 1 hour followed by washing for 3 times. For preparation of RBCs fluorescently labeled with CytoRed, 1 × 10^7^ mouse RBCs were incubated in 100 μl of DMEM medium containing 50 μg/ml of CytoRed at 4°C for 30 minutes, and then washed 3 times with DMEM.

### *In vitro* hemophagocytosis assay

BM cells were cultivated on 8-well chamber slides (Thermo Fisher) at a density of 1 × 10^6^ cells/ml. The Day 6 BMDMs were transfected with si-Control or si-ATP6V0D2 for 24 hours. On Day 7, the silenced BMDMs were infected with *L. donovani* promastigotes at MOI of 20 and incubated for 72 hours. The BMDMs were then incubated with CytoRed-labeled RBCs for 2 hours. Cell nuclei were counterstained with Hoechst33342 and counting of hemophagocytes was performed using BZ-X810 microscope (Keyence, Osaka, Japan).

### *Leishmania* intracellular survival assay

BMDMs treated with either si-Control or si-ATP6V0D2 were infected with *L. donovani* at MOI of 20 and the extracellular parasites were washed off 24 hours post infection. Cells were incubated for additional 48 hours and then fixed with methanol for 5 minutes and applied for Giemsa staining. The number of intracellular amastigotes of over 100 macrophages from each group was calculated, and the means of three independent experiments were used in calculation of infection index. The infection index was defined as the ratio of the amastigote number in silenced group against the amastigote number per cell in non-silenced *L. donovani* infection group. To verify whether ATP6V0D2 silencing affects the initial invasion of the parasites into macrophages, both of the treated BMDMs were infected with *L. donovani* for 6 hours. After washing off the extracellular parasites with PBS, the cells were fixed with methanol and applied for Giemsa staining.

To examine whether the existence of RBCs in macrophages affects intracellular survival of *Leishmania* in an ATP6V0D2-dependent manner, BMDMs either left untreated or pretreated with either si-Control or si-ATP6V0D2 for 24 hours were incubated with opsonized RBCs for 2 hours at 37°C. After removal of extracellular RBCs by lysing with RBC lysing buffer for 2 minutes, the BMDMs were infected with *L. donovani* at MOI of 20 and the extracellular parasites were washed off 24 hours post infection. Cells were incubated for another 48 hours and then fixed with methanol and applied for Giemsa staining.

For iron chelation in the BMDMs with opsonized RBC supplementation, after removal of extracellular RBCs, BMDMs were incubated with 10 μM of deferiprone (DFP: Wako, Japan) before and during infection with *L. donovani*.

### Giemsa staining

BMDMs cultivated on chamber slides were fixed with methanol for 5 minutes and stained with 5% Giemsa solution (Sigma) diluted in distilled water for 20 minutes. After air drying, the slides were rinsed with xylene and then mounted in Mount quick (Daido Sangyo, Japan).

### Labile iron pool analysis

To explore whether ATP6V0D2 affects the concentration of intracellular labile iron pool in *L. donovani*-infected cells, calcein-acetoxymethyl ester (Calcein-AM) staining was performed. Calcein AM is a cell-permeable dye that can be used to measure cell viability. In living cells, non-fluorescent Calcein-AM is converted to green fluorescent calcein after hydrolysis of the acetoxymethyl ester by intracellular esterases. Iron binding to calcein quenches its fluorescence, which can be recovered by diminishing its iron by a strong chelator, and therefore fluorescence by calcein staining is thought to negatively correlate with the amount of labile iron pool in cytoplasm (Soe-Lin et al., 2006). BMDMs treated with either si-Control or si-ATP6V0D2 were incubated with opsonized RBCs for 2 hours and infected with CytoRed-labelled *L. donovani* for 6 hours after the incubation. The cells were incubated with 1 μM of Calcein-AM (Dojindo Laboratories) in PBS for 1 hour at 37°C.

The fluorescence intensity of the stained cell images was analyzed using BZ-X800 analyzer software (Keyence). The green fluorescence channel images were used for analyzing the light brightness of each visual field.

### Quantitative RT-PCR

Day 6 BMDMs were treated with either si-Control or si-ATP6V0D2 for 24 hours and supplemented with opsonized RBCs for 2 hours. RNA was extracted using TRIzol reagent (Invitrogen) following the manufacturer’s instructions. The concentration of total RNA was measured by DU730 Life Science UV/vis spectrophotometer (Beckman Coulter, USA). 4 μg of total RNA was used as a template for synthesis of 20 μl of cDNA. A tube containing 500 ng oligo (dT)16, and 10 nmol dNTPs (Fisher Scientific, UK) with template RNA was incubated for 5 minutes at 65˚C at a 13 μl reaction volume. After adding 5× first strand buffer, 200 nmol DTT (Thermo) and 200 U of M-MLV (Thermo), the tube was incubated at 37°C for 50 minutes. The reaction was inactivated by incubation for 15 minutes at 70°C. The synthesized cDNA was used for expression analyses of murine ferroportin (*Fpn*), heme oxygenase 1 (*Hmox1*), transferrin receptor 1 (*Tfrc*) and β-actin (*Actb*). Primers used in this study were listed in **Supplementary Table 1**.

Real-time PCR assay was conducted using 1 μl reverse transcription PCR product as the template and 10 μl of SYBR Select Master Mix on the ABI Prism 7000 Sequence Detection System. Data were analyzed by 2-ΔΔCt methods through normalization with murine *Actb*. The thermal cycling conditions were 94°C for 10 minutes, followed by 40 cycles at 94°C for 15 seconds and 60°C for 1 minute.

### Statistical analysis

Statistical comparisons were performed by one-way ANOVA followed by Tukey’s multiple comparisons test or unpaired t test with GraphPad Prism 9 software. A difference between groups was considered as statistically significant when the *P* value was less than 0.05.

## Results

### Knockdown of *Atp6v0d2* reduces the formation of hemophagocytic MGCs in *L. donovani* infected BMDMs

**Figure 1:**
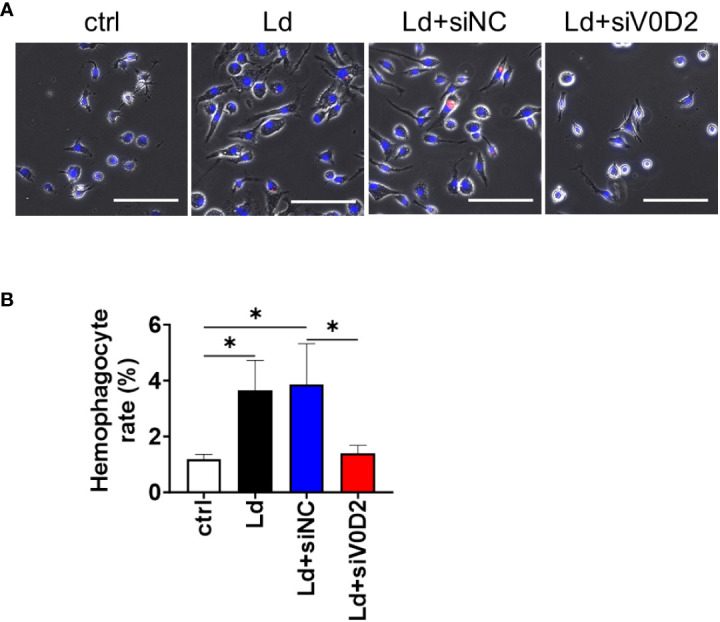
Hemophagocytosis rate of *Atp6v0d2*-knockdown *L. donovani* infected BMDMs. BMDMs were transfected with either siRNA of *Atp6v0d2* or random negative control 24 h before *L. donovani* infection. The BMDMs were incubated with fresh murine erythrocytes and examined for hemophagocytosis. **(A)** Representative images of BMDMs engulfing CytoRed-labeled RBCs with Hochest33342 counterstaining. Bars, 100 μm. **(B)** The proportion of hemophagocytes in each group is shown. Means + SD of three independent experiments are shown. ns, no significance, *P < 0.05, by one-way ANOVA followed by Tukey’s multiple comparisons test.

In the previous study, we demonstrated that multinucleation of macrophages is induced by *L. donovani* infection via upregulation of ATP6V0D2 (Hong et al., 2022). Here, an association between the upregulation of ATP6V0D2 with the hemophagocytic feature of MGCs was examined. Expression of *Atp6v0d2* in BMDMS was upregulated by *L. donovani* infection and the induction was canceled by *Atp6v0d2* siRNA but not with control siRNA as previously reported (Hong et al., 2022). Along with that, *L. donovani*-infected BMDMs exhibited higher frequency to engulf untreated erythrocytes, whereas the upregulated hemophagocytosis was canceled by *Atp6v0d2* siRNA but not with control siRNA (**Figure 1**).

### Upregulated ATP6V0D2 expression supports intracellular survival of *L. donovani* in the presence of erythrocyte supplementation

**Figure 2:**
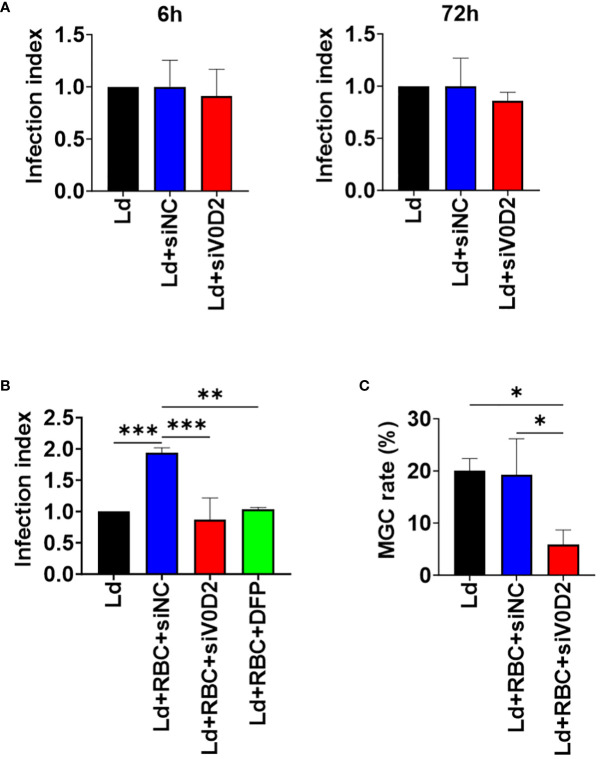
Altered intracellular survival of *L. donovani* in *Atp6v0d2*-knockdown BMDMs with or without opsonized erythrocyte supplementation. BMDMs were transfected with either si-control or si-Atp6v0d2 siRNA for 24 hours before *L. donovani* infection. The number of intracellular amastigotes was counted in over 100 BMDMs. The infection index refers to the ratio of the number of intracellular amastigotes per BMDM in each group to that in sole *L. donovani* infection group. **(A)** Infection indexes of si-control or si-Atp6v0d2-treated BMDMs without opsonized RBC supplementation at 6 hours and 72 hours post initial infection. **(B)** BMDMs were incubated with opsonized RBCs for 2 hours before *L. donovani* infection. One group of cells was infected and supplemented with opsonized RBCs as the other groups while being additionally treated with DFP. Counting of intracellular amastigotes were performed at 72 hours of infection and infection indexes were calculated as described earlier. **(C)** BMDMs were incubated with opsonized RBCs for 2 hours before *L. donovani* infection. The proportion of cells with 2 or more nuclei in over 100 cells at 72 hours post infection is shown. Means + SD of three independent experiments are shown. *P < 0.05, **P<0.01, ***P<0.001 by one-way ANOVA followed by Tukey’s multiple comparisons test.

First, to examine the effect of upregulated ATP6V0D2 to intracellular survival of *L. donovani* within macrophages in the absence of erythrocyte supplementation, *Atp6v0d2*-knockdown BMDMs were infected with *L. donovani*. When examined at 6 or 72 hours of infection, no significant difference in the number of intracellular parasites between the control group and *Atp6v0d2*-knockdown group was observed (**Figure 2A**).

Next, to simulate the microenvironment of spleen and bone marrow in which infected macrophages are surrounded by erythrocytes enabling hemophagocytosis, both the control cells and *Atp6v0d2*-knockdown cells were first supplemented with opsonized RBCs and then infected with *L. donovan*i. Knockdown of *Atp6v0d2* did not affect internalization of opsonized RBCs by BMDMs (**Supplementary Figure 1**). Intracellular parasite load in BMDMs increased in the presence of opsonized RBCs (**Figure 2B**), and this promotional effect on intracellular survival by opsonized RBC supplementation was canceled by *Atp6v0d2* knockdown (**Figure 2B**). To see an involvement of iron in this beneficial effect of RBC supplementation, BMDMs supplemented with opsonized erythrocytes were treated with an iron chelator DFP and then infected with *L. donovani.* The increased intracellular parasite survival was diminished, and the infection index became equivalent to that of BMDMs without RBC supplementation as well as that of BMDMs with *Atp6v0d2* knockdown (**Figure 2B**). Infection-induced MGC formation was also suppressed in *L. donovani-*infected BMDMs with *Atp6v0d2* knockdown (**Figure 2C**).

### Knockdown of *Atp6v0d2* increases cytosolic labile iron pool in *L. donovani*-infected BMDMs supplemented with opsonized RBCs

**Figure 3:**
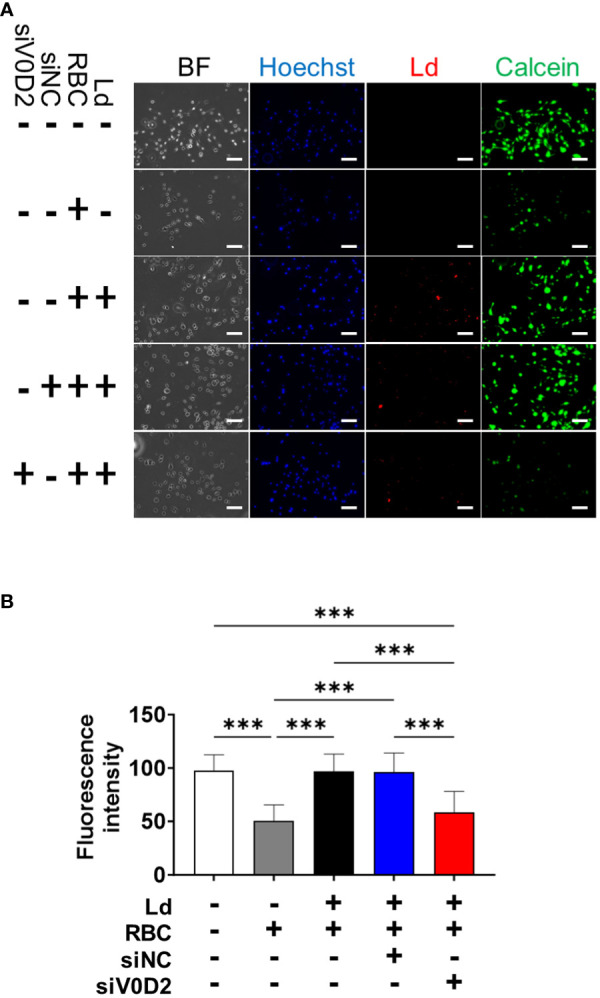
Increased cytosolic labile iron pool level in *Atp6v0d2*-knockdown *L. donovani* infected BMDMs. **(A)** BMDMs were transfected with either si-control or si-Atp6v0d2 for 24 h before infection with CytoRed-labeled *L. donovani*. At 6 hours post infection, BMDMs were incubated with opsonized erythrocytes for 2 hours. Then the cells were stained with calcein-AM and counterstained with Hoechst33342. BF, Bright field. Bars, 100 μm. **(B)** Fluorescence intensity of calcein-AM stained cells per visual field. Means + SD of over 3 visual fields in each group are shown. ***P < 0.001 by one-way ANOVA followed by Tukey’s multiple comparisons test.

To examine whether ATP6V0D2 affects labile iron pool within infected, RBC-supplemented BMDMs, a calcein-AM staining-based assay was performed. Supplementation with opsonized RBCs caused a decrease in fluorescence intensity by calcein staining (**Figure 3**). Fluorescent intensity was high when RBC-supplemented BMDMs treated with control siRNA were infected with *L. donovani* (**Figure 3**). On the other hand, fluorescent intensity was low when RBC-supplemented BMDMs treated with *Atp6v0d2* siRNA were infected with *L. donovani* (**Figure 3**).

## Discussion

ATP6V0D2 is previously characterized as an inducer of macrophage multinucleation in *Leishmania* infection (Hong et al., 2022). Here we reported that the elevated expression level of this protein is also associated with hemophagocytosis activity in the infected macrophages. Because hemophagocytosis and multinucleation are closely connected in splenic macrophages infected with *L. donovani in vivo* (Morimoto et al., 2016), it is not surprising to see that knockdown of *Atp6v0d2* in *L. donovani*-infected BMDMs inhibited not only MGC formation but also hemophagocytosis in these cells (**Figure 1**). In fact, MGC formation itself is thought to be induced to change phagocytic characters of myeloid cells. For example, IL-4 induces the formation of foreign body giant cells supposedly to engulf larger material which is hardly phagocytosed by mononuclear macrophages (Milde et al., 2015). Therefore, ATP6V0D2 may contribute to enhanced phagocytosis of RBCs during *L. donovani* infection at least by inducing MGC formation and making them easier to engulf extrinsic materials. On the other hand, not all the MGCs induced by distinct mechanisms are hemophagocytic; in fact, BMDMs treated with GM-CSF + IL-4 were not hemophagocytic while MGC formation being induced by stimulation with those cytokines (Hong et al., 2022). Although whether ATP6V0D2 engages in self-cell recognition or inhibitory signaling for self-cell phagocytosis remains elusive, mechanisms involving the molecule other than just MGC formation may play roles in induction of *L. donovani*-induced hemophagocytosis.

Knockdown of *Atp6v0d2* did not affect the number the intracellular parasites either at 6 hours or 72 hours post infection of *L. donovani* (**Figure 2A**), which is concordance with the report on *L. amazonensis*-infected BMDMs (Pessoa et al., 2019). It suggests that ATP6V0D2 does not directly affect initial internalization of the parasites or their intracellular survival in the absence of hemophagocytosis. Although it is reported that lysosomal ATP6V0D2 promoted Yes-associated protein (YAP) lysosomal degradation and substantially enhance IFN-β production (Shen et al., 2021), and IFN-β is thought to sustain intracellular survival of *L. donovani* (Dias et al., 2022), we did not observe upregulated IFN-β expression accompanied with ATP6V0D2 expression in transcriptomic analysis (data not shown). By contrast, when macrophages were supplemented with opsonized RBCs, ATP6V0D2 contributed to increased parasite survival within macrophages (**Figure 2B**). Therefore, it is suggested that the ATP6V0D2-mediated benefits to intracellular survival of *L. donovani* are not dependent on MGC formation itself but involves hemophagocytosis prominent in *Leishmania*-induced MGCs. In fact, we found that ATP6V0D2 is involved in processing of labile iron pool supplied from phagocytosed RBCs (**Figure 3**). Our results demonstrated that cytosolic iron concentration in macrophages increases following engulfment of RBCs whereas the increase is canceled by *L. donovani* infection. We suppose the labile iron is delivered into phagolysosome and consumed by the parasites, since the cytosolic labile iron is one of the major sources of iron to intracellular amastigotes (Goto et al., 2023).

Erythrocyte processing in hemophagocytic macrophages is associated with the release of heme from phagolysosomes into the cytoplasm via assistance of heme responsive gene-1 (HRG1) (Rajagopal et al., 2008). Heme accumulation in the cytoplasm induces the expression of heme oxygenase-1 (Hmox1), a heme-catabolizing enzyme that extract iron (Gozzelino and Soares, 2014). The resulting iron is either excreted from macrophages via the transmembrane protein ferroportin (FPN), or is stored intracellularly by combination with ferritin (Knutson et al., 2005), unless otherwise delivered to and consumed in the other organelles. It is reported that V-ATPase-mediated lysosomal acidification is essential for regulating cellular iron traffic (Milde et al., 2015). Several reports supported that inhibition of V-ATPase activity impairs release of lysosomal iron into cytosol (Yambire et al., 2019; Hughes et al., 2020). Despite ATP6V0D2 is generally considered to be expressed as a component of V-ATPase and locates in lysosomes (Xia et al., 2019), the majority of *Leishmania*-induced ATP6V0D2 does not locate in lysosomes (Hong et al., 2022). In fact, due to the existence and broad expression of its isoform *Atp6v0d1*, which is possibly partial redundant to *Atp6v0d2* (Pietrement et al., 2006), lysosome acidification is not sharply affected even in macrophages deficient of *Atp6v0d2* (Pessoa et al., 2019). In addition, the expression of *Hmox1*, the enzyme that catalyzes the degradation of heme group, was not affected by *Atp6v0d2* knockdown (**Figure S2**). Therefore, at least the release of RBC-derived heme from phagolysosome into cytosol or the release of iron from the heme in cytosol may not be affected by ATP6V0D2. It is important to experimentally elucidate whether the released iron in cytosol is substantially transferred into *Leishmania*-containing phagolysosome and utilized by the parasites for explaining the increased cytosolic iron in *Atp6v0d2*-knockdown infected macrophages. Quantification of the iron concentration in isolated *Leishmania*-containing phagolysosomes (Banerjee and Datta, 2020) may provide us a new insight to learn the detailed mechanisms of ATP6V0D2 in iron transportation.

It is demonstrated in *L. amazonensis* infection that the removal of FPN from plasma membrane stimulates parasite proliferation (Ben-Othman et al., 2014). Inhibition of FPN translation in *L. donovani*-infected macrophages leads to higher intracellular parasite burden (Das et al., 2018). We also compared the expression of FPN and transferrin receptor 1 that regulate iron transportation in *L. donovani*-infected BMDMs in the presence/absence of *Atp6v0d2* knockdown, whereas no significant difference was observed between the groups (**Supplementary Figure 2**). Together, upregulated iron export from the cell and inhibition of other iron importers are unlikely to explain ATP6V0D2-mediated loss of labile iron pool in RBC-supplemented, *L. donovani*-infected macrophages. Of course, expression of FPN on plasma membrane is not only regulated transcriptionally but is rather mediated by internalization triggered by hepcidin (Nemeth et al., 2004). Therefore, further analyses are required to understand iron trafficking in this experimental condition.

It raises the possibility that ATP6V0D2 is associated with the utilization of cytosol ferrous iron either by ROS-producing mitochondria or intracellular parasites, although it remains to be proven experimentally by comparing the expression of mitochondrial iron transporter mitoferrin (*Slc25a37*) (Shaw et al., 2006) with other vacuolar iron transporters like ATPase cation transporting 13A2 (*Atp13a2*) (Zhang et al., 2022). With the beneficial outcome of hemophagocytosis to the intracellular parasites, iron transport to parasitophorous vacuoles and iron consumption by the parasites also need to be considered as possible events mediated by ATP6V0D2. As forementioned, internalization of FPN is triggered by hepcidin (Nemeth et al., 2004). Following the internalization process, FPN mainly localizes in early endosome and lysosome (Sabelli et al., 2017) which directly consist or have close contact with *Leishmania* parasitophorous vacuoles. Furthermore, it is also worthy notice that hepcidin is secreted by macrophages and it shares a common transcription factor TFEB with *Atp6v0d2* (Sow et al., 2007; Matsumura et al., 2022). It suggests that these molecules are expressed parallelly to facilitate the endosomal/lysosomal transportation of free iron and benefit *Leishmania* parasites for survival within hemophagocytic macrophages.

## Data availability statement

The raw data supporting the conclusions of this article will be made available by the authors, without undue reservation.

## Ethics statement

The animal study was approved by the Animal Experiment Committee at the University of Tokyo. The study was conducted in accordance with the local legislation and institutional requirements.

## Author contributions

JH: Data curation, Formal analysis, Investigation, Methodology, Validation, Visualization, Writing – original draft. BM: Conceptualization, Investigation, Methodology, Writing – review & editing. CS: Methodology, Resources, Writing – review & editing. JY: Data curation, Formal analysis, Investigation, Methodology, Validation, Writing – review & editing. YG: Conceptualization, Data curation, Formal analysis, Funding acquisition, Investigation, Methodology, Resources, Supervision, Validation, Visualization, Writing – review & editing.
