# Supplementary material for: Upregulation of ATP6V0D2 benefits intracellular survival of Leishmania donovani in erythrocytes-engulfing macrophages
Source: Front Cell Infect Microbiol. 2024 Jan 31;14:1332381. doi: 10.3389/fcimb.2024.1332381 (PMC10864549; doi:10.3389/fcimb.2024.1332381)
Supplement: Supplementary file 1 [file DataSheet_1.docx]

**
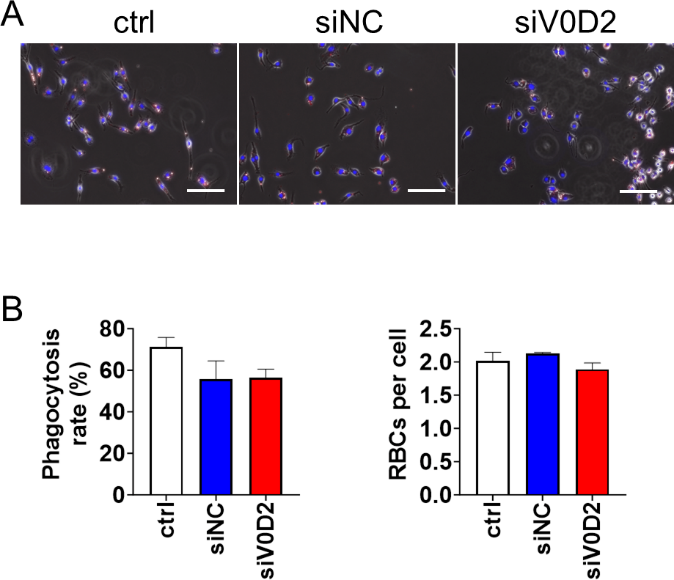
**

**Figure S1. Equivalent phagocytosis of opsonized RBCs by *Atp6v0d2*-knockdown BMDMs.** (A) Representative images of BMDMs engulfing opsonized CytoRed-labeled RBCs. Cell nuclei were stained with Hoechst 33342. Bars, 100 μm. (B) Proportion of BMDMs engulfing opsonized RBCs (left) and the average number of phagocytosed RBCs per BMDM (right) in each group are shown. Comparison was performed by one-way ANOVA followed by Tukey’s multiple comparison test with no significant difference among the groups.

**
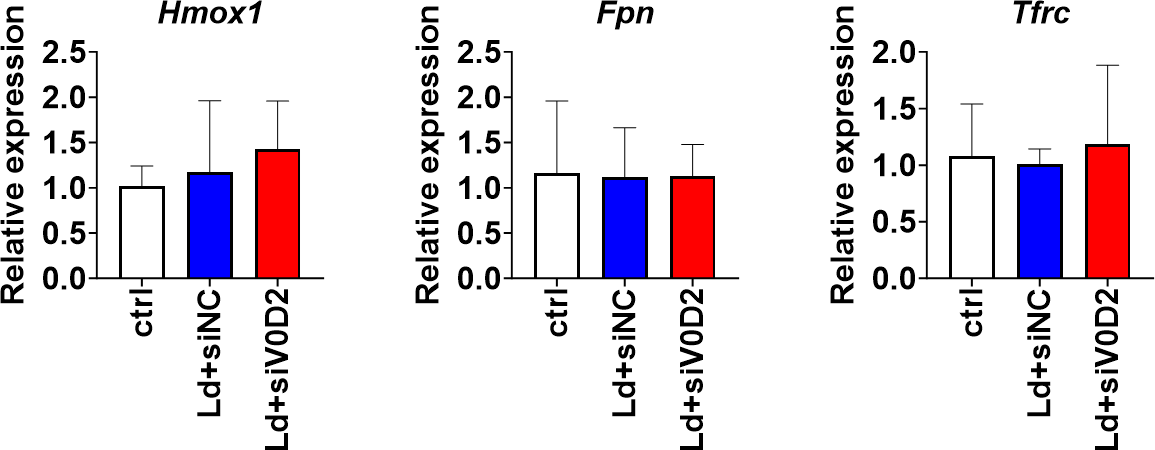
**

**Figure S2. Equivalent expression of iron-related genes in *L. donovani-*infected BMDMs with or without silencing *Atp6v0d2.*** BMDMs were transfected with either siRNA of *Apt6v0d2* or random negative control for 24 hours before infection with *L. donovani*. Total RNA was isolated from BMDMs at 24 hours after infection. Relative expressions of *Hmox1*, *Fpn*, *Tfrc* mRNA to *Actb* mRNA were quantified by qPCR. Comparison was performed by unpaired t-test with no significant difference between the groups.

**Table S1. Primers used in this study**

| Primers | Sequences |
| --- | --- |
| *Actb*-forward | GTTACCAACTGGGACGACA |
| *Actb*-reverse | TGGCCATCTCCTGCTCGAA |
| *Fpn*-forward | CTACCATTAGAAGGATTGACCAGCT |
| *Fpn*-reverse | ACTGGAGAACCAAATGTCATAATCTG |
| *Tfrc*-forward | TCATGAGGGAAATCAATGATCGT |
| *Tfrc*-reverse | GCCCAGAAGATATGTCGGAA |
| *Hmox1*-forward | CACGCATATACCCGCTACCT |
| *Hmox1*-reverse | CCAGAGTGTTCATTCGAGCA |
